# Relationship between Parental Feeding Practices and Neural Responses to Food Cues in Adolescents

**DOI:** 10.1371/journal.pone.0157037

**Published:** 2016-08-01

**Authors:** Harriet A. Allen, Alison Chambers, Jacqueline Blissett, Magdalena Chechlacz, Timothy Barrett, Suzanne Higgs, Arie Nouwen

**Affiliations:** 1 School of Psychology, University of Nottingham, Nottingham, United Kingdom; 2 School of Psychology, University of Birmingham, Nottingham, United Kingdom; 3 Department of Experimental Psychology, University of Oxford, Oxford, United Kingdom; 4 NIHR Wellcome Clinical Research Facility, Birmingham Children’s Hospital, Birmingham, United Kingdom; 5 Department of Psychology, Middlesex University, London, United Kingdom; McMaster University, CANADA

## Abstract

Social context, specifically within the family, influences adolescent eating behaviours and thus their health. Little is known about the specific mechanisms underlying the effects of parental feeding practices on eating. We explored relationships between parental feeding practices and adolescent eating habits and brain activity in response to viewing food images. Fifty- seven adolescents (15 with type 2 diabetes mellitus, 21 obese and 21 healthy weight controls) underwent fMRI scanning whilst viewing images of food or matched control images. Participants completed the Kids Child Feeding Questionnaire, the Childrens’ Dutch Eating Behaviour Questionnaire (DEBQ) and took part in an observed meal. Parents completed the Comprehensive Feeding Practices Questionniare and the DEBQ. We were particularly interested in brain activity in response to food cues that was modulated by different feeding and eating styles. Healthy-weight participants increased activation (compared to the other groups) to food in proportion to the level of parental restriction in visual areas of the brain such as right lateral occipital cortex (LOC), right temporal occipital cortex, left occipital fusiform gyrus, left lateral and superior LOC. Adolescents with type 2 diabetes mellitus had higher activation (compared to the other groups) with increased parental restrictive feeding in areas relating to emotional control, attention and decision-making, such as posterior cingulate, precuneus, frontal operculum and right middle frontal gyrus. Participants with type 2 diabetes mellitus also showed higher activation (compared to the other groups) in the left anterior intraparietal sulcus and angular gyrus when they also reported higher self restraint. Parental restriction did not modulate food responses in obese participants, but there was increased activity in visual (visual cortex, left LOC, left occipital fusiform gyrus) and reward related brain areas (thalamus and parietal operculum) in response to parental teaching and modelling of behaviour. Parental restrictive feeding and parental teaching and modelling affected neural responses to food cues in different ways, depending on motivations and diagnoses, illustrating a social influence on neural responses to food cues.

## Introduction

Social context is an important influence on eating behaviour. How much people eat and what they choose to eat depends on who they dine with [1]. For instance, adults tend to eat more when eating with friends and family than when alone but, on the other hand, tend to eat less in the company of strangers or other people they wish to impress [1]. In addition, people tend to follow social norms when making food-related decisions even if other people are not present at the time of decision making [2]. For example, exposing a person to information that others are consuming the recommended amount of vegetables in their diet results in greater selection of vegetables at a lunch buffet than when they are simply exposed to the health benefits of eating vegetables [3]. Children and adolescents are similarly susceptible to the influence of social context on eating [4], in particular, the influence of their parents. Parental feeding practices affect the eating environment, food availability and learning about foods, which have all been related to variability in food responsiveness and tendency towards overeating [5].

Several types of parental feeding practice have been previously identified including controlling practices, emotional and instrumental feeding and modelling [6, 7]. Some parents are reported to attempt to use foods to either manage children’s mood state (emotional feeding) or to reward desirable behaviour (instrumental feeding)[8]. These practices have been associated with increased responsiveness to food cues. Controlling feeding practices, involving for example restricting access to “unhealthy” foods, are often imposed by parents of obese children due to concerns about their children’s weight [9] [10] but are not effective in improving the quality of the diet or reducing adiposity in the long term. On the contrary, these feeding practices lead to increased adiposity through their negative impact on children’s eating behaviour. Perceptions of parental controlling feeding practices are associated with greater dietary self restraint, emotional and external eating in preadolescents [11], extreme weight control behaviours [12] and unhealthy eating attitudes [13] in adolescent samples. Controlling feeding practices have also been associated with higher food responsiveness and eating in the absence of hunger [14] and greater emotional eating [15]. Restrictive control appears to be particularly deleterious in its effects on subsequent weight gain in samples of children at risk for being overweight on the basis of parental adiposity [16]. Teaching about eating, on the other hand, especially in the context of modelling intake of foods by parents, has been associated with lower food fussiness and food responsiveness [17]. For example, children are much more likely to try a new food if they see a parent also eating that same food [18].

The use of parental feeding practices in young people vary depending on individual child characteristics, such as their weight. For example, parents of overweight or obese adolescents report greater use of restriction and monitoring than the parents of lean adolescents [19, 20]. Parents who are anxious about their child's eating and weight are more likely to use restrictive feeding practices [10]. A factor that has received little attention is how feeding practices and their consequences relate to the motivations underlying these practices. Restriction of eating may arise from a concern about weight gain but may be required for medical reasons, for example to ensure that a child is following a prescribed diet for control of type 2 diabetes [21]. However, little is known about how parental feeding practices interact with such motivations to influence adolescent eating responses. The specific mechanisms underlying the effects of parental feeding practices on their children’s eating are also unknown. It has been reported that social influence more generally affects activity in brain areas associated with the neural computation of the subjective value of rewards [22, 23]. One possibility is, therefore, that parental feeding practices affect reward-related responses to food cues and this explains at least in part subsequent behaviour around food. Based on this evidence, it is possible that parental feeding practices of restriction, emotive feeding and teaching will be associated with eating behaviour via an effect on neural processes related to food reward.

Recent neuroimaging studies suggest a strong link between obesity and activation of reward related circuits [24]. It has been proposed that obesity, and by extension type 2 DM, results from distortions in reward saliency, motivation (towards food), learning and inhibitory control (e.g. [25]). Consistent with this, several studies have found increased activation to food cues in reward related areas such as caudate, insula, or orbitofrontal cortex in adults with obesity [26]. Furthermore, the activation level of these areas seems to be modulated by context such as stress [27] in obese, but not lean, adults. Although no studies have investigated the social or familial context of eating behaviour in adolescents, it has been found that emotional eating modulates food cue responses in the insula for obese adults (40–70 years old) and in the insula, orbitofrontal cortex and amygdala for adults with type 2 DM [28]. In younger adults, self-restraint in eating has been found to be related to modulation of the functional connectivity in temporal visual association networks (occipital and fusiform regions) [29].

Here we examine how variations in parents’ feeding practices link to adolescents’ responsiveness to food cues using fMRI and a measure of eating in the absence of hunger. We predicted that the use of restriction, emotive feeding and teaching practices will be associated with enhanced reactivity to food cues in reward-related brain areas. In addition, we examine whether similar relationships hold for the parental practices of children for whom there may be specific motivations to control the diet due to weight control (obesity) or medical reasons (type 2 diabetes mellitus; type 2 DM); specifically, perhaps the effect of parental control on reactivity to food cues and reward might be less detrimental when control is motivated by medical reasons rather than weight control goals alone.

While traditionally confined to the middle-aged and elderly population, there has been a marked world-wide increase in type 2 diabetes among younger people [30, 31]. Although its causes are likely to be multi-factorial, childhood obesity is believed to be an important underlying factor [31]. Progression from prediabetes to full diagnosis can be much quicker in the young than in the older population [32]. Both obesity and type 2 DM have been associated with increased activity in brain areas linked to reward when cued to, or reminded of, food [8, 33, 34]. We hypothesise that after diagnosis, adolescents with type 2 diabetes and their carers, who will have received specific dietary counselling and medical care, will show differential feeding practices. This, then, will alter the social context of food. We were, thus, able to examine whether enhanced reward related responding in obesity and type 2 DM are related to specific parental feeding practices.

## Methods

### Participants

There were 57 adolescent participants, including fifteen with type 2 DM, 21 obese and 21 healthy weight controls (see Table 1). Adolescents with type 2 DM were referred to us by paediatric endocrinologists in the UK Midlands and North-West within the duration of the project. Selection criteria included: (*1*) between 12–18 years, (*2*) being able to understand and read English and (*3*) diagnosis of type 2 DM > 6 months. Obese adolescents were referred by dieticians or responded to advertisements and were included if their BMI exceeded defined International Obesity Task Force age specific cut offs [35]. Healthy weight control participants were recruited from local schools. Exclusion criteria were; major medical conditions (except diabetes, polycystic ovarian syndrome, hirsutism) or learning disabilities, claustrophobia, contraindications to MRI and major changes in diabetes related medication within 6 months. To confirm group allocation, within a 6-month period, before or after scanning, all control and obese participants had their fasting glucose, c-peptide and HbA1c measured and underwent an oral glucose tolerance test. For participants with diabetes clinical data were obtained from their medical files. Clinical and demographic data were obtained from the participants’ clinical files or collected during this study. The groups were matched for (self reported) ethnicity and recruited from similar geographic areas. Fully informed written consent was taken from all participants and their respective parent/guardian prior to participation. The study protocols were approved by the National Research Ethics Committee and the Birmingham University Imaging Centre. Two participants were excluded from analysis, not reflected in the numbers above, due to signal loss and a brain abnormality.

**Table 1:** Demographics and relevant histories of participants, by group. Age differences were controlled for in the analysis.

Characteristic	Type 2 diabetes mellitus^a^ (N = 15)	Obese^b^ (N = 21)	Healthy weight controls^c^ (N = 21)	F	df	p	Results Tukey Post-hoc Tests
Age (years)	16.08 ± 1.53	14.89 ± 1.98	16.0 ± 1.91	2.88	2,54	0.065	ns
BMI (SD)	2.16 ± 1.51	3.19 ± 0.81	0.25 ± 1.01	38.30	2,54	< .001	C<T2DM<O
HbA_1c_ (%, sd, mmol/mol, n)	8.26 ± 2.26, 67 (n = 14)	5.55 ± 0.38, 37 (n = 20)	5.29 ± 0.32, 34(n = 17)	27.80	2,48	< .001	T2DM>C,O
Fasting blood glucose	9.92 ± 3.92 (n = 12)	4.91 ± 0.523 (n = 21)	4.80 ± 0.48 (n = 20)	33.16	2,53	< .001	T2DM>C,O
Duration of diabetes (months)	35.8 ± 30.7	NA	NA				
Diabetes treatment	(n)	(n)					
Tablets (Metformin)	7	4	NA				
Insulin	3	NA	NA				
Tablets+Insulin	1	NA	NA				
GLP-1	1						
GLP-1+Tablets	1						

Values are means ± SD

^a^ n = 15: female only

^b^ n = 20: 15 female

^c^ n = 20: 14 female, T2DM = type 2 disbetes mellitus.

A note on sample size: Chechlacz et al. (2009) found that the smallest between groups contrast for food images was found for the insula (Z = 2.79), which corresponds with a Cohen’s d of 1.17. Using an alpha of 0.5 and power of 0.80 the minimum required sample size for the fMRI study was 13 participants per group. Hill et al. (2008, International Journal of Obesity, vol. 32, no. 10, pp. 1499–1505.) examined eating in the absence of hunger in 9–12 year olds and found an effect size for comparison of two groups of .52. Therefore with 3 groups, an alpha of .05, and power of .95, the ideal sample size is 63.

### Stimuli

**Fig 1:**
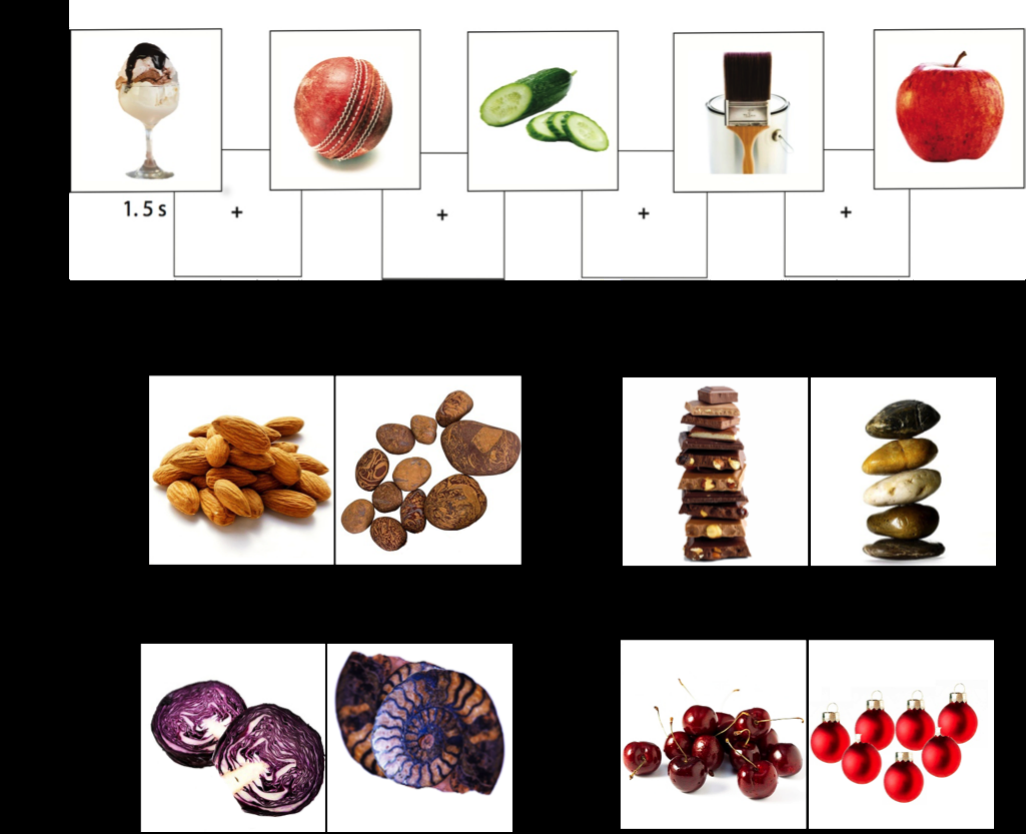
a) illustration of extract of stimulus sequence. b) examples of stimulus and control pairs from each condition.

A total of 120 food and 120 non-food visually matched (in shape, complexity, brightness and colour) control pictures and 1 target picture were used (Fig 1). The non-food pictures were unrelated to food e.g. buttons, furniture. The food pictures included equal amounts of high fat, high sugar foods (e.g. cake, ice-cream); high fat, low sugar foods (e.g. fried chicken, nuts); low fat, high sugar (e.g. sweets, apples); low fat, low sugar foods (e.g. carrots, peas).

### Procedure

Participants were asked to consume their usual breakfast at home, at approximately 8am, or arrived at 8am and were given breakfast (cereal, juice, toast). All adolescents were then asked to complete the following questionnaires: the Kid’s Child Feeding Questionnaire (KCFQ), to examine perceived parental feeding practices [36], the Perceived Deprivation Scale [37], the Children’s Dutch Eating Behaviour Questionnaire (DEBQ-C) to examine their external, emotional and restrained eating styles [38], and dietary self-efficacy [39] and the adapted motivation for dietary self-care [40, 41]. A parent/guardian was asked to complete the adult version of DEBQ, and the Comprehensive Feeding Practices Questionnaire (CFPQ) [42]. Adolescents with type 2 DM were also asked to complete the Summary of Diabetes Self-Care Activities scale [43] and the Diabetes Responsibility Scale [21]. Participant weight and height was measured while wearing light clothes without footwear to allow calculation of BMI. Height was measured with a Seca 213 portable stadiometer. Weight was measured with Tanita 384 digital scales. BMI was calculated and converted to z scores adjusted for age and gender according to British 1990 growth reference (UK90) curves [44].

Participants' current disposition to eat (hunger, thirst and fullness) was measured using a10cm Visual Analogue Scale, from 0 (not at all) to 10 (very much). There were no differences between the groups on pre-scan hunger, thirst and fullness scores (F(2, 55) = .205 p = .82; F(2,55) = 1.409 p = .23; F(2,55) = .166 p = .89).

Immediately, prior to the scanning session, at 10.30am, blood glucose was measured by the finger prick method, using a FreeStyle Optium Blood Glucose Monitor. Where necessary, participants were encouraged to take their normal medication or consume a small snack.

#### fMRI

Participants viewed food and non-food pictures within the MRI scanner. Pictures were presented using an event-related design in a pseudo random order. Each picture was presented for 1.5s and followed by a fixation cross (3.5s to 9s). Each picture was presented once and the experiment was split into 3 separate runs. Participants were instructed to look carefully at each picture and to imagine eating the food item. Participants were asked to press a button when they saw a yellow smiley face, presented five times per run at random intervals.

After exiting the scanner, participants then rated the food pictures that were shown to them during the scan on how appetising they thought the food was on scale from 1 (not appetising) to 7 (very appetising) and also their current desire to eat each food from 1 (not at all) to 7 (very much).

#### Post-scanning

After the scanning session both adolescent and parent were taken to a behaviour-recording suite. Participants were invited to have lunch, sat at a table and were each given a tray containing a set prepared meal consisting of: cheese sandwich (150g, 370kcal), individual Chicago Town cheese pizza (150g, 453kcal), bowl of cherry tomatoes (50g, 18kcal), Activia strawberry yoghurt (125g, 123kcal), Granny Smith apple (188kJ, 45kcal), satsuma (approx 75kJ, 18kcal), Walkers ready salted crisps (25g, 130kcal), two Maryland double chocolate cookies (22g, 106kcal) and jug of water and glasses. They were asked not to share food from each other's trays and told that they were not expected to eat all the food, but to eat until they were full. After the participants indicated they had finished, the parent was asked to leave the room to complete further questionnaires and the adolescent was asked to wait. A tray with 4 bowls filled with Maltesers (50g, 253kcal), cucumber (80g, 253kcal), grapes (100g, 60kcal) and Hula-Hoops (25g, 127kcal) were placed in front of the adolescent together with books, magazines and food rating scales. The adolescent was told they could eat the snacks, whilst they waited, and was left for 10 minutes. Food was weighed before and after eating. The researcher left the room during the meal and snack. A remotely adjustable Sony camera (EVI-D70) recorded the eating behaviour.

### fMRI acquisition

Participants were scanned on a Phillips 3T Achieva MRI scanner with 8-channel phased array SENSE head coil. Functional scans were acquired using a blood oxygen level-dependent (BOLD) contrast weighted echo planar sequence (TR = 2500ms, TE = 35ms, 82° flip angle, 4 slices, 2.5x2.5x3mm voxels, 96x96 pixel matrix). A T1-weighted image was acquired for co-registration and display of functional data (TR 8.4ms, TE 3.8ms, flip angle 8°, matrix resolution 288x288, 175 slices, 1x1x1mm voxels).

### fMRI Analysis

FMRI data processing used FMRI Expert Analysis Tool Version 5.98, part of FSL (FEAT, www.fmrib.ox.ac.uk/fsl). The following pre-statistics processing was applied; motion correction using MCFLIRT [45]; slice-timing correction using Fourier-space time-series phase-shifting; non-brain removal using BET [46]; spatial smoothing using a Gaussian kernel of FWHM 5mm; grand-mean intensity normalisation of the entire 4D dataset by a single multiplicative factor and highpass temporal filtering. Melodic ICA was used to identify and remove artefacts from the data. FILM was used for time series statistical analysis with prewhitening [47]. Registration of functional to high resolution and standard images was done using FLIRT [45].

Responses to food cues were evaluated in a whole brain analysis. The first level (within scan) design matrix included regressors for the food, non-food and target trials and motion related regressors. Scans were averaged first within participants and then between groups. Age and gender were entered as covariates of no interest, in addition, to check whether gender affected our results, we re-ran the analysis with only females and found qualitatively the same results. For the reported analyses, Z (Gaussianised T/F) statistic images were thresholded using clusters determined by Z>2.3 and a (corrected) cluster significance threshold of P = 0.05 [48]. To test whether responses to food cues were modulated by behavioural measures (i.e. restriction, emotional feeding etc, see below) these were added as regressors to the general linear model at the group stage. Specific group comparisons are described in the results section.

### Factor Analysis

To generate parsimonious variables for describing feeding and eating behaviour, factor analyses of the feeding and eating behaviour variables were conducted for data reduction purposes before examining relationships between the psychological and neuro-imaging variables. Responses from the CFPQ, DEBQ and KCFQ were combined to create factors for parental behaviour. Responses from the DEBQ-C, dietary self-efficacy and the adapted motivation for dietary self-care were analysed to create factors describing adolescent behaviour.

## Results

### Behavioural Measures

A series of ANOVAs were used to compare measures between groups (see Tables 1 and 2). Each adolescent’s BMI was converted to a Z score (SD-BMI) based on the British 1990 growth reference for height, weight, and body mass index (Cole, Freeman and Preece, 1995). Obese adolescents were defined as having a SD-BMI exceeding 1.96 standard deviations from the mean (>95th percentile). Results are reported for analysis without controlling for education but pattern of results is the same if this is controlled. Participant groups differed in terms of age as well as BMI and diabetes related measures, as expected (Table 1). They did not differ on any other characteristics. There were small group differences in dietary self-efficacy, and adapted motivation for healthy diet measures with the control group scoring highest on the self-efficacy measure and those with obesity scoring higher for amotivation (Table 2). Both the adolescents with type 2 DM and their parents ate the least sugar at lunch, other than this there were no overall significant differences between the groups for the amount eaten (Table A and Table B in S1 File), or in overall hunger or thirst ratings. The ratings of desirability and appeal of the food images did not differ.

**Table 2:** Questionnaire measures for eating style and reported desirability of the food images. The table shows the results of comparisons between the group. Significance values reflect results of one way ANOVAs between groups.

Measure	type 2 DM^a^	Obese^b^	Healthy weight controls^c^	F	p
DEBQ-C					
Emotion	1.50 ± 0.59	1.39 ± 0.58	1.31 ± 0.44	0.544	0.584
Restraint	2.16 ± 0.43	1.90 ± 0.61	1.65 ± 0.55	3.873	0.027*
External	1.86 ± 0.49	1.78 ± 0.64	1.88 ± 0.47	0.194	0.825
KCFQ					
Pressure	0.54 ± 0.38	0.54 ± 0.38	0.59 ± 0.40	1.607	0.210
Restriction	0.96 ± 0.30	1.09 ± 0.32	0.60 ± 0.27	14.076	< .001**
Perceived deprivation scale	153.67 ± 26.27	153.65 ± 46.96	153.50 ± 43.00	0.000	1.000
Motivation for healthy diet					
Identified	4.40 ± 0.78	4.07 ± 0.88	4.04 ± 0.73	1.038	0.361
Extrinsic	2.66 ± 0.83	2.61 ± 1.06	2.40 ± 0.82	0.379	0.686
Amotivation	1.87 ± 0.78	2.13 ± 0.87	1.39 ± 0.65	4.596	0.015*
Intrinsic	3.74 ± 0.97	3.44 ± 0.81	3.26 ± 0.82	1.329	0.274
Self-efficacy healthy diet	46.52 ± 14.14	45.20 ± 18.48	58.86 ± 12.69	4.580	0.015*
CFPQ					
Child control	2.99 ± 0.60	2.98 ± 0.69	3.17 ± 0.78	0.450	0.640
Emotional regulation	1.64 ± 0.87	1.57 ± 0.54	1.55 ± 0.68	0.086	0.918
Balance and variety	4.32 ± 0.63	4.45 ± 0.69	4.31 ± 0.63	0.168	0.846
Environment	4.02 ± 0.66	3.70 ± 0.71	3.88 ± 0.88	0.754	0.476
Reward	2.00 ± 1.15	2.13 ± 1.04	1.70 ± 0.90	0.902	0.412
Involvement	3.84 ± 1.18	4.25 ± 0.87	3.82 ± 1.04	1.082	0.347
Modelling	3.92 ± 0.84	4.01 ± 0.71	3.91 ± 0.88	0.094	0.911
Monitoring	4.38 ± 0.76	4.08 ± 0.67	3.37 ± 0.84	8.328	0.001**
Pressure	2.27 ± 0.66	2.17 ± 0.88	2.65 ± 1.02	1.643	0.203
Restriction health	3.83 ± 0.98	4.16 ± 0.76	3.18 ± 1.05	5.307	0.006**
Restriction weight	3.73 ± 0.85	3.43 ± 0.81	2.47 ± 0.90	10.75	< .001**
Teaching	4.12 ± 0.59	4.05 ± 0.70	4.00 ± 0.81	0.125	0.883
DEBQ					
Emotion	2.46 ± 0.82	2.76 ± 0.57	2.68 ± 0.60	0.940	0.397
Restraint	3.26 ± 0.69	3.04 ± 0.75	2.82 ± 1.09	1.089	0.344
External	2.00 ± 1.06	2.32 ± 0.89	2.01 ± 0.84	0.761	0.472
Picture ratings					
Appeal					
HFHS^d^	4.53 ± 1.43	4.71 ± 1.60	4.34 ± 1.17	0.318	0.729
HFLS^e^	3.95 ± 0.98	4.01 ± 1.60	3.98 ± 1.05	0.011	0.989
LFHS^f^	4.17 ± 1.39	4.36 ± 1.16	4.94 ± 1.02	2.098	0.133
LFLS^g^	3.29 ± 1.00	3.16 ± 1.41	3.56 ± 1.08	0.598	0.554
Desire to eat					
HFHS^d^	2.66 ± 1.74	3.02 ± 1.91	2.64 ± 1.55	0.294	0.746
HFLS^e^	2.48 ± 1.19	2.64 ± 1.60	2.60 ± 1.44	0.060	0.942
LFHS^f^	2.85 ± 1.51	3.20 ± 1.61	3.80 ± 1.52	1.700	0.193
LFLS^g^	1.97 ± 1.01	1.87 ± 1.39	2.38 ± 1.29	0.920	0.405
Hunger Ratings					
Before lunch	58.8 ± 25.6	54.4 ± 19.7	57.8 ± 10.4	.197	.822
After lunch	14.5 ± 17.6	14.9 ± 16.7	10.4 ± 13.2	.500	.609

Values are means ± SD

^a^ n = 15: female only

^b^ n = 20: 15 female, 5 male

^c^ n = 20: 14 female, 6 male

^d^ High fat, high sugar

^e^ High fat, low sugar

^f^ Low fat, high sugar

^g^ Low fat, low sugar

*p<0.05

**p<0.001.

The factor analyses revealed 3 factors each for the parent and adolescent behaviours (Table C and Table D in S1 File). Parental behaviours loaded on to factors that we labelled Teaching and Modelling (discussion of foods, promoting variety and modelling behaviours), Emotive Feeding (using food to modulate emotion, or as reward) and Restrictive Feeding (monitoring and guiding food intake and the child's perception of this). Adolescent questionnaire responses loaded on to three similar factors labelled Self Efficacy (perception of their own ability to control their diet), Emotion/External regulation (the desire to eat when emotional, in the presence of palatable food or other people) and Self Restraint (controlling food to avoid weight gain).

For healthy weight controls and obese participants, none of our factors correlated significantly with eating behaviour or intake (Table E in S1 File). Teaching and Modelling was negatively associated with snack intake for participants with type 2 DM (Table E in S1 File).

### fMRI Results

**Fig 2:**
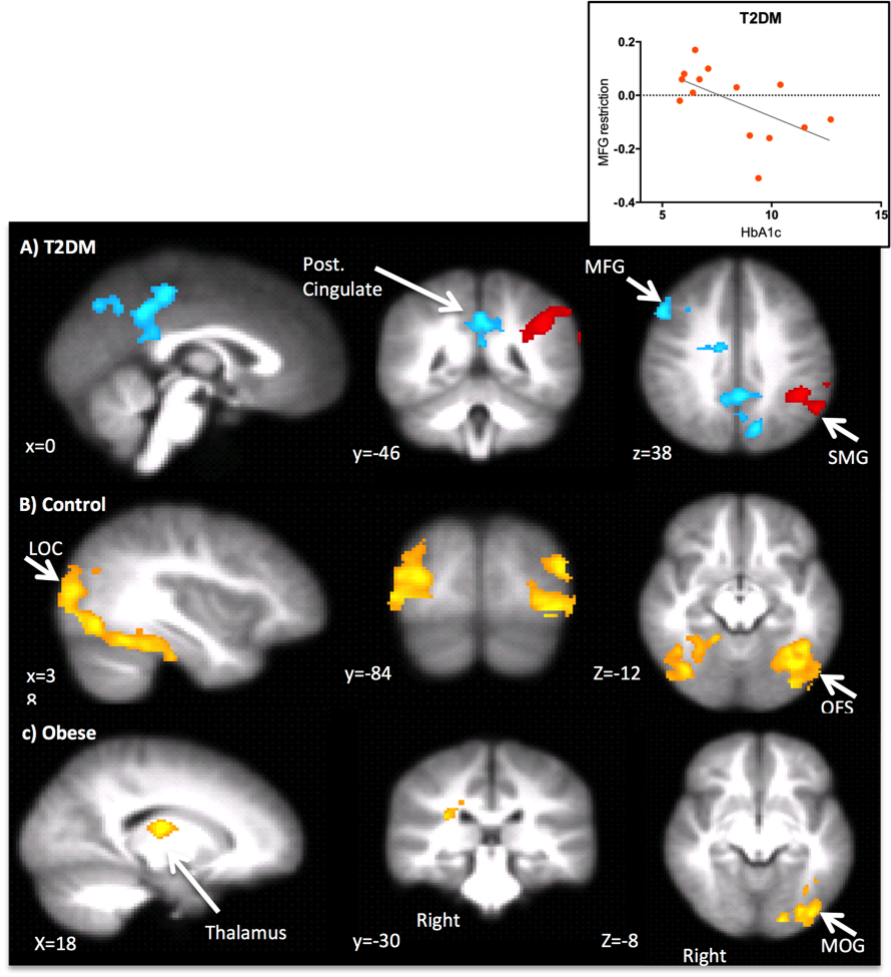
**a) Areas where activation for Restrictive Feeding (blue) and Self Restraint (red) was significantly higher for the Type 2 diabetes mellitus group compared to the other groups. b) Areas where there was significantly greater activation related to Restrictive Feeding in the control group compared to the other groups. c) Areas where there was significantly greater activation related to Teaching of Healthy Eating in the obese group, compared to other groups**. Activations are shown on the average brain for the study. Insert: Relationship between HbA1c test value and MFG modulation to restriction. MFG = Middle Frontal Gyrus, SMG = Supramaginal Gyrus, LOC = Lateral Occipital, OFS = Occipital Fusiform Gyrus, MOG = Medial Occipital Gyrus/occipital cortex.

**Fig 3:**
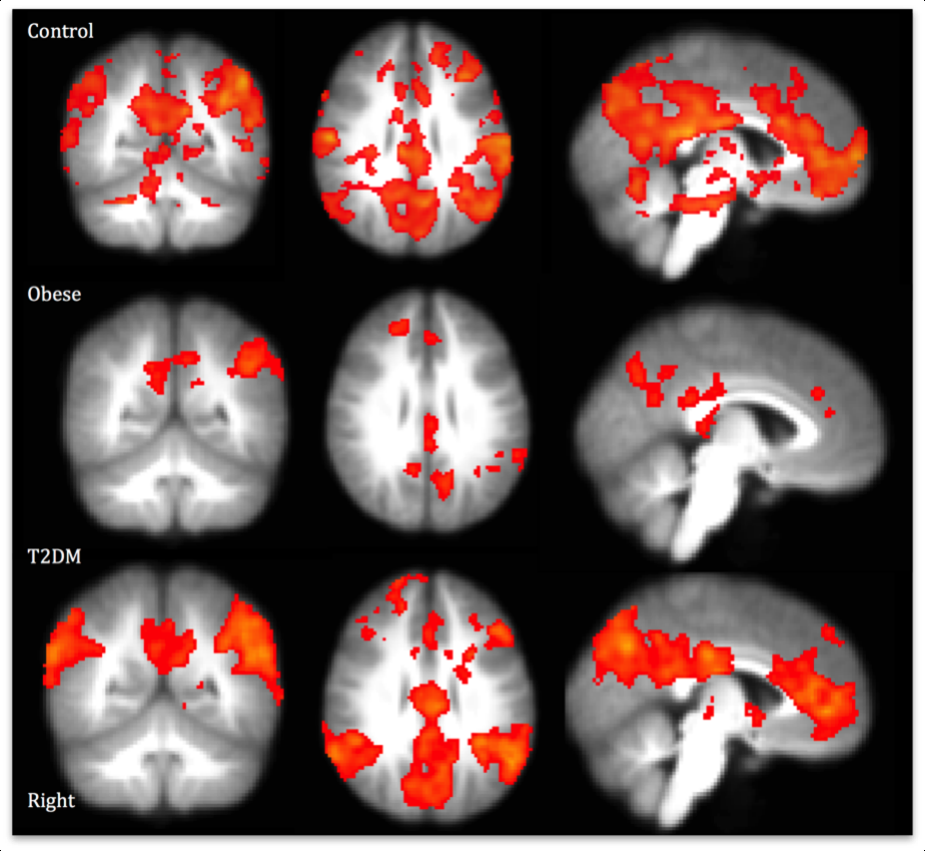
Areas of the brain where there is higher (cluster corrected p<0.05, Z>2.3) activation for food images compared to visually matched non-food images for the three groups. Note that there was no significant difference between the groups when these were directly contrasted so this figure should be interpreted with caution. Colour scale is from red (z score = 2.3) to yellow (z score = 6.6). All images are shown at 2.3–6.6, x = -4, y = -54, z = 30 (MNI). Activations are shown on the average brain for the study. Activations for the three groups overlapped at the right frontal pole, left and right anterior and posterior cingulate, left supramarginal Gyrus, and in the precuneus (mostly left).

The factors above were used as regressors in the analysis of fMRI data to find areas that were responsive to food images and modulated by feeding or eating behaviour (Fig 2, Tables 3 and 4). To facilitate comparison with existing literature we also report results of the contrast of food images vs. non food images without modulation by these factors (Fig 3, Table F in S1 File). We report the results from analysis of all participants but the pattern of results was not different when we analysed only girls (self-defined by participants), or only those without polycystic ovary syndrome (n = 5).

**Table 3:** Food related areas that were more active in participants with reported higher *parental* Restrictive feeding and Teaching and Modelling. The table shows areas that were significantly active in the groups alone as well as areas that were found to be significantly more active in one group contrasted with the other groups, indicated in the left column. IPS: Intra Parietal Sulcus. WM: White matter.

Contrast/ Factor	Size (vox)	Z MAX	Peak & sub- peak MNI co-ordinates			Region
			X	Y	Z	
**Restrictive Feeding**						
Healthy weight control mean	2585	4.63	-20	-90	-2	Left occipital pole
		4.58	-26	-92	-2	Left occipital pole
		4.16	24	-92	0	Left occipital pole
		4.12	-38	-86	-8	Left lateral occipital cortex
		3.85	40	-50	-20	Right temporal occipital fusiform
		3.83	-4	-100	8	Left occipital pole
T2 Diabetes mean	2889	4.36	-2	-50	40	Precuneus
	2397	4.12	54	-24	-14	Right middle temporal gyrus
			42	2	-18	Right planum polare
	1936	4.29	-46	-28	-12	WM/Left middle temporal gyrus
			-36	18	-18	Left orbital frontal
			-68	-40	14	Left superior temporal gyrus
	1256	3.7	8	54	2	Right paracingulate
			-4	34	12	Anterior cingulate
	1183	4.1	-4	28	60	Left superior frontal gyrus
Healthy weight controls > T2 Diabetes + Obese	2817	4.22	40	-72	-6	Right lateral occipital cortex
			42	-56	-20	Right temporal occipital fusiform
	2660	4.79	-36	-64	-10	Left occipital fusiform gyrus
			-40	-64	-8	Left lateral occipital cortex
			-42	-86	22	Left superior lateral occipital cortex
T2 Diabetes > Obese + Healthy weight controls	1242	3.73	-4	-40	44	Posterior cingulate gyrus
			-2	-36	50	Precuneus cortex
	610	3.23	24	22	20	WM/Frontal Operculum
			52	22	36	Right middle frontal gyrus
** • Teaching and Modelling**						
Obese mean	625	4.47	-28	-34	48	Left somatosensory cortex
			-34	-30	44	Left somatosensory cortex
			-24	-48	50	Left superior parietal lobule
	618	4.25	18	-14	16	Right thalamus
			36	-38	22	Right Parietal Operculum
Obese > Healthy weight controls+ T2 Diabetes	745	3.65	-16	-90	-8	Left visual cortex
			-40	-82	-8	Left lateral occipital cortex
			-18	-86	-12	Left occipital fusiform gyrus
	622	4.05	34	-38	22	Thalamus, Parietal Operculum

**Table 4:** Food related areas that were more active in participants with reported higher *self* emotional/external regulation, self restraint and self-efficacy. The table shows areas that were significantly active in the groups alone as well as areas that were found to be significantly more active in one group contrasted with the other groups, indicated in the left column.

Contrast/Factor	Size (Vox)	Z Max	Peak & sub- peak MNI co-ordinates			Region
			X	Y	Z	
**emotional/external regulation**						
Obese mean	739	4.43	26	22	-6	Right putamen
		3.75	20	4	2	Right putamen
		3.63	34	-6	0	Right insula
		3.46	48	-12	-10	Right superior temporal gyrus
		3.26	10	-2	-2	Right thalamus
** • Self-Restraint**						
T2 Diabetes mean	1486	4.66	-44	-46	36	Left anterior IPS
		3.94	-56	-56	40	Left angular gyrus
	755	3.9	62	-54	30	Right angular gyrus
	681	3.73	6	-76	48	Precuneus
T2 Diabetes -obese-control	1141	4.29	-44	-46	36	Left anterior IPS
		3.53	-44	-54	54	Left angular gyrus
**Self-Efficacy**						
Healthy weight controls	1810	4.06	32	56	2	Right frontal pole
	988	4.05	44	-54	56	Right angular gyrus

### Modulation of food cue activations

There was greater activity for healthy weight controls, compared to those with obesity and type 2 DM, to food images relating to Restrictive Feeding in posterior (predominantly visual) regions of the brain (Fig 2B, Table 3). Teaching and Modelling was associated with increased activity for obese participants (compared to the other groups) to food images in visual brain areas, the thalamus and the parietal operculum (Fig 2, Table 3).

There was greater activity compared to the other groups to food images relating to Restrictive Feeding in the adolescents with type 2 DM in the posterior cingulate, precuneus and middle frontal gyrus (MFG) compared to the other participants (Fig 2, Table 3). Restrictive Feeding related activation in the MFG was negatively correlated with HbA1c in this group, suggesting that when there is Restrictive Feeding, increased frontal activation to food is associated with better diabetes control. There was also unique activation for those with type 2 DM in relation to adolescents' own reports of Self Restraint in the intraparietal sulcus and the angular gyrus (Table 4).

### Responses to food images vs. non food images without modulation by behaviour

For comparison with the literature we also report areas where there was higher activation for food images, compared to non-food images, for each group separately. There were no significant differences between the groups for activation to food (compared to non-food) images. Healthy weight participants showed extensive activation to food compared to non-food images (Fig 3, Table F in S1 File). There were peaks in gustation-related areas and activations in areas typically associated with food and reward, such as insula and operculum [49, 50]. In obese participants, food images (compared to non-food images) activated regions in the left precuneus, thalamus, left supramaginal and angular gyrus and regions around the frontal pole and superior frontal gyrus (Fig 3, Table F in S1 File). As expected, participants with type 2 DM had higher activation to food, compared to non-food images, without accounting for behaviour, in an extensive network of brain areas (Fig 3, Table F in S1 File). The regions found were similar to those previously reported to be responsive to food cues and associated with gustation and reward [50].

Activation for food images was correlated with BMI in the type 2 DM and obese groups in a range of regions including the left superior frontal gyrus, left and right inferior frontal gyri, left precuneus, left cingulate and right medio-temporal gyri (Table G in S1 File). For the healthy weight and obese participants, BMI modulated responses to food cues in a similar set of frontal regions, but also the operculum and cingulate.

Activation for images of greater fat food content was higher for people with type 2 DM than for those with obesity in a range of predominantly visual or visual assocation or attention areas (Table H in S1 File) including the calacrine sulcus, right termporal gyrus, right angular gyrus and inferior parietal regions. Activation for high fat food was greater in healthy weight controls than in people with obesity in regions including the left and right insula, left and right operculum and around the right supramarginal gyrus and left inferior frontal gyrus.

## Discussion

We investigated the differences in eating styles and behaviour between control (healthy weight) adolescents and adolescents who were obese or had type 2 diabetes. We show that parental feeding practices had different associations with brain responses to food among different groups of adolescents. We found that the three groups demonstrated different responses to food that could not be captured by observational, behavioural or brain imaging results alone. Healthy weight participants showed higher visual activation to food cues when there was higher Restrictive Feeding. For adolescents with obesity the Teaching and Modelling factor was related to higher brain activity in visual and reward related areas. Participants with type 2 DM showed unique food related brain activations modulated by parental Restrictive Feeding practices. This is despite only small differences in food judgements or food consumed (although it should be noted that this was a single recorded eating episode in a laboratory setting). We show, therefore, that parental behaviours are associated with food responses in their children and that this depends on individual differences in motivations to control diet.

We hypothesised that parental restriction would be associated with enhanced reactivity to food cues in reward related areas. Consistent with this, for those with type 2 DM at least, Restrictive Feeding increased activity in the precuneus and posterior cingulate. The areas partially overlap with areas implicated in hedonic hunger [51] this might be consistent with increased reward response to food. These areas, however, are not consistent with more conventional reward or food motivation driven areas. For instance, they do not overlap with areas implicated in the anticipation of chocolate milk reward or that are moderated by treatments known to moderate food reward such as glucagon-like peptide 1[52].

Brain responses to different eating and feeding practices showed different relationships in groups of adolescents believed to have specific motivations to control the diet. Those with a diagnosis of type 2 DM might be considered to have the highest motivation to improve their diet and will have received advice from medical professionals. Consistent with this, the type 2 DM group scored highest on the motivation for dietary change questionnaire and were higher on the Monitoring and Restriction questionnaire measures. For this group there were clear differences in brain activity modulations in response to Restrictive Feeding (see above). In contrast, Restrictive Feeding did not modulate brain activity in those with obesity. For the healthy weight controls, Restrictive Feeding increased activation in brain regions specialised for vision consistent with increased attentional weight (or ‘saliency’) for restricted food rather than reward per se. This suggests that the pattern of activity for those with type 2 DM was linked specifically to the increased motivation for dietary change. This perhaps suggests a mechanism by which behavioural effects of parental restriction have different effects depending on children's tendency to overeat [53].

We expected that the influence of parental feeding would depend on diagnosis (or lack of it). Consistent with this, for obese participants (without diagnosis with type 2 DM), higher scores on the Teaching and Modelling factor, but not Parental Restriction, were related to higher brain activity in visual and reward related areas. This occurred despite the obese group not being significantly higher than the other groups on teaching and modelling related questionnaire scores. This illustrates that within this group, activity is modulated by these parental behaviours, even if the overall level of these behaviours is not higher. Consistent with our results, activation to both food cues and learnt rewards has previously been found to be higher in the parietal operculum in obese adolescents [54] but we extend this to relate specifically to parental teaching. In the obese participants, even Teaching and Modelling may be linked to a heighted orienting of attention to food and linking of food with reward. Indeed, weight change after a weight loss intervention has been found to be positively correlated with food related activation in both reward and visual areas i.e. more activation in visual areas was correlated with weight gain, rather than loss [55].

We found this relationship between teaching and enhanced reward only existed in the obese group. Our data do not allow us to establish the direction of this relationship, nor examine the content or manner of teaching used by parents, so it is possible that the obese adolescents’ focus and attention to food elicits parental teaching about eating. It is worth noting, however that some of our obese group is likely to already have impaired glucose metabolism and that we lack data on weight changes prior to the study. This is almost inevitable in such a sample since the progression for obese adolescents to type 2 diabetes is known to be faster than in adult groups [32]. It is also possible that there is a qualitative difference in the type of teaching received by obese adolescents in comparison to adolescents who are healthy weight or who have type 2 DM that could not be captured by the measures used in this study. Even with these caveats, it is possible to say that parental feeding practices influence neural responses to food differentially, depending on diagnosis.

As well as differences in motivations towards food, the three groups received different medical treatments. Most participants with type 2 DM were receiving Metformin, Insulin, GLP-1 or some combination of the three. Four (of 21) obese participants were receiving Metformin. This study was not designed to investigate the differences between medications, so conclusions about the differential effects cannot be drawn here. Nevertheless it should be noted that insulin has been proposed to have a protective effect in the brain against cognitive decline, at least in older people [56, 57]. Furthermore, insulin administered to young people over 8 weeks has been shown to improve memory and performance on a set of cognitive tests [58]. Similarly there is some evidence that metformin treatment (in rats) improves performance on memory-based tasks [58]. It is possible that the medication received by those with a diagnosis of type 2 DM (or obesity) improves their ability to develop appropriate coping mechanisms or feeding behaviours, as well as acting directly on metabolism.

This study cannot fully elucidate the relationship between Restrictive Feeding and food responses in type 2 DM, but it is worth noting that activity in one of the areas activated, the MFG, was positively correlated with diabetes control (i.e. HbA1c). This area has been implicated in emotional control [59] suggesting that adolescents may be starting to automatically invoke control mechanisms. This is also consistent with our previous finding in older people with type 2 DM that inhibiting emotional responses to food supports dietary management [34]. Alternatively, those with better diabetes control may then have more cognitive resources to support dietary control [60]. Longitudinal studies are necessary to disambiguate this question.

Adolescents with type 2 DM own reports of Self Restraint were also linked to increases in activity in parietal regions commonly linked to spatial attention [61]. These areas are also within the postulated temporal visual association network, activation within which has been correlated with self restraint scores in young adults [29]. This is consistent with both parental Restrictive Feeding and Self-Restraint leading to more complex processing of food cues perhaps involving consideration of duties and obligations [62] and decision making [63] all of which might be consistent with participants’ responses to external restriction of food. Restriction in those with type 2 DM leads to intense processing of food cues, perhaps connected to emotional control, duties or decision-making, despite minimal differences in observed behaviour. This is in contrast to the healthy weight controls where Restrictive Feeding was related to increases in brain regions associated with visual processing, consistent with simple increases in saliency.

### Unmodulated responses to food cues

Several studies have suggested that people with T2DM or obesity show alterations in striatal regions (e.g. Nucleus Accumbens, caudate, putamen) limbic (amygdala, hypothalamus, thalamus) and cortical areas such as the insula or inferior parietal cortex in response to food cues or images [64, 65]. It is proposed that obesity increases the anticipatory reward to food cues (but reduces the reward of food itself) [25]. Although we did find responses to food cues in a set of similar areas to those found previously, including parts of the striatal and limbic systems, in the three groups (Table F in S1 File), we did not find the expected between group differences. Furthermore, although activity in frontal and medial areas (i.e. precuneus and cingulate) correlated with BMI (Table G in S1 File), we did not find correlations between food cue responses and activation in striatal or limbic areas. This suggests that in our groups, at least, the proposed reward pathways to simple presentation of food images, are not yet clearly discriminable between groups.

Previous studies have suggested that people with obesity, and, where tested, type 2 DM also show higher activations to images of high calorie food, particularly in the regions described above [64]. This subject has been studied extensively; see [50] for a review. For instance, in a group of obese women, high- calorie food images (compared to low calorie images) activated the putamen [66]. On the other hand, an analysis restricted to reward related regions of interest found that obese women had higher activation in a range of areas (e.g. orbitofrontal regions, insula, anterior cingulate, caudate, hippocampus) but not the putamen [67]. Contrary to these previous findings, we found lower activation for obese, compared to healthy weight participants, in operculum, insula, inferior and orbitofrontal cortex for high fat foods (Table H in S1 File), suggesting, counterintuitively, lower anticipatory reward responses. Those with type 2 DM show higher activation (compared to obese) in predominantly visual or visual association areas, suggesting greater salience of food cues in the diabetic group. Thus, our results, whilst replicating typical gustation reward activations, do not seem to show the same pattern of effects of obesity or type 2 DM.

Chechlacz et al. (34) found that activations in the orbitofrontal cortex and left insula were not only positively associated with external eating and predicposition to eat, but also to dietary self-efficacy and dietary self-care in adults with T2DM. This suggests that, in adults, these areas might be involved restraint immediate desire in favour of long-term outcomes. The data presented here imply that this function may be impaired in the obese group.

One important difference between our participants and those in previous studies is their age. There is some evidence that responses to food cues change with age, at least with healthy weight participants. It has previously been reported that whilst (healthy weight) adolescents show activation to food images in predominantly visual areas (e.g. fusiform gyrus), adults show activation in prefrontal cortex, and it has been suggested that there is an increased role of prefrontal regions in the processing of food with age [50, 68]. There have been few studies of brain activation to food cues in obese adolescents, but what evidence there is suggests that there is little difference in responses to food cues between obese adolescents with, and without insulin resistance, although there were differences in reward related brain activation between lean and obese participants [64]. In our groups, activations are consistent with increasing reward and saliency for only the participants with the highest BMI. It is possible that the documented reward pathways to food take some years to develop, but that the restraint, restriction and teaching behaviours above facilitate and shape these pathways.

### Conclusions

We demonstrate that different motivations towards food lead to both different brain activation patterns and different modulations of brain responsivity to food cues. Those with high motivations to improve their diet show neural modulation in proportion to the degree of parental restriction. Parental Teaching and Modelling, on the other hand, was linked to increases in activation in reward related areas. We conclude that social and external factors can influence food based brain activity in the absence of intake and eating behaviour differences.

## Supporting Information

S1 File (PDF)
