# Supplementary material for: Relationship between Parental Feeding Practices and Neural Responses to Food Cues in Adolescents
Source: PLoS One. 2016 Aug 1;11(8):e0157037. doi: 10.1371/journal.pone.0157037 (PMC4968823; doi:10.1371/journal.pone.0157037)
Supplement: S1 File — (PDF) [file pone.0157037.s001.pdf]

Supporting Information for
Allen et al

Table A: Amounts of food consumed during the observed lunch for both the parent/carer and child.

Meal Consumption	Type 2 DM[a]	Obese[b]	Healthy weight controls[c]	F	p
Adolescent					
Calories (Kcal)	837.00 ± 249.33	911.45 ± 265.98	839.53 ± 205.92	0.569	0.570
Protein (g)	29.56 ± 9.60	30.84 ± 10.83	28.73 ± 8.71	0.229	0.796
Carbohydrate (g)	89.08 ± 24.42	102.80 ± 26.97	96.76 ± 23.43	1.233	0.300
Sugar (g)	20.86 ± 11.51	29.84 ± 11.88	33.39 ± 11.89	4.692	0.014*
Fat (g)	39.70 ± 12.96	41.22 ± 12.92	36.76 ± 10.21	0.688	0.507
Parent					
Calories (Kcal)	655.41 ± 225.88	651.25 ± 254.57	703.03 ± 272.44	0.240	0.787
Protein (g)	23.56 ± 8.90	25.24 ± 10.90	26.74 ± 11.58	0.374	0.690
Carbohydrate (g)	67.72 ± 26.81	68.18 ± 25.08	78.27 ± 25.04	0.998	0.376
Sugar (g)	15.46 ± 9.66	26.62 ± 10.77	28.33 ± 10.52	7.364	0.002**
Fat (g)	32.23 ± 13.07	30.35 ± 12.81	31.42 ± 15.23	0.088	0.916

Values are means ± SD
[a] n= 14: female only
[b] n= 20: 15 female, 5 male
[c] n= 19: 14 female, 5 male

Table B: Amounts of food consumed by the adolescents when presented with snacks away from the presence of their carer/parent. Adolescents were told that they could take as much or as little as they liked of the snacks whilst they waited for their parent to complete another part of the study.

Snack Consumption	Type 2 DM[a]	Obese[b]	Healthy weight Controls[c]	F	p
Adolescent					
Calories (Kcal)	42.37 ± 72.48	88.19 ± 127.48	53.47 ± 63.52	1.149	0.325
Protein (g)	0.57 ± 0.99	1.06 ± 1.48	0.72 ± 0.93	0.800	0.455
Carbohydrate (g)	6.11 ± 10.51	13.10 ± 18.74	9.85 ± 12.42	0.934	0.400
Sugar (g)	4.74 ± 9.12	10.55 ± 14.70	6.84 ± 9.07	1.135	0.330
Fat (g)	1.71 ± 3.05	3.56 ± 5.44	2.55 ± 4.07	0.738	0.483

Values are means ± SD
[a] n= 14: female only
[b] n= 20: 15 female, 5 male
[c] n= 19: 14 female, 5 male

Table C Factors and loadings for exploratory factor analysis on *parent* behaviour. Extraction method used was PCA with varimax-rotation (an orthogonal rotation of component axes). KMO = 0.660 Bartlett's Sphericity = p<0.001 Total variance explained 53.73% Comprised of: Teaching =18.7% Emotive feeding = 17.69% Restriction =17.34%. %. Subscales with loadings < 0.4 were excluded from the factor analysis, these included KCFQ pressure and CFPQ child control subscales.
The table shows the abbreviated questionnaire name and subscale and how this loaded to the factors used in the fMRI analysis.
Examples of questions loading on to the Teaching and Modelling Factor: ' I discuss with my child the nutritional value of foods.', 'I encourage my child to try new foods.'.
Examples of Questions loading on to Emotive Feeding: 'When this child gets irritable, is giving him/her something to eat or drink the first thing you do?', 'I withhold sweets/dessert from my child in response to bad behavior.'
Examples of questions loading on to the Restrictive Feeding Factor: 'How much do you keep track of the high-fat foods that your child eats?', 'If I did not guide or regulate my child's eating, s/he would eat too much of his/her favorite foods.'

	Teaching and Modelling	Emotive feeding	Restrictive Feeding	Communalities
DEBQ-emotional	-0.364	**0.753**	-0.093	0.709
DEBQ-external	-0.342	**0.723**	0.117	0.653
CFPQ-teaching nutrition	**0.556**	0.193	0.097	0.355
CFPQ-modelling	**0.768**	0.224	0.151	0.662
CFPQ-environment	**0.54**	-0.366	0.134	0.443
CFPQ-emotional regulation	0.171	**0.77**	0.035	0.624
CFPQ- balance and variety	**0.715**	-0.08	0.062	0.522
CFPQ-pressure	0.383	**0.438**	-0.409	0.506
CFPQ-food reward	0.176	**0.504**	0.14	0.304
CFPQ-monitoring	0.334	-0.227	**0.648**	0.506
CFPQ-restriction health	0.195	0.213	**0.785**	0.7
CFPQ-restriction weight	0.35	0.327	**0.599**	0.589
KCFQ-restriction	-0.009	0.207	**0.73**	0.576
KCFQ-general restriction	-0.023	-0.155	**0.636**	0.429

Table D Factors and loadings for exploratory factor analysis on *adolescent* questionnaire scores. Extraction method used was PCA. KMO = 0.636 Bartlett's Sphericity = p<0.001 Total variance explained 65.42% Comprised of: Emotion/external regulation =22.90% Self restraint = 21.91% Self efficacy =20.61%. The Perceived Deprivation Subscale was removed due to the measure of sampling adequacy being < 0.5.
The table shows the abbreviated questionnaire and subscale and how this loaded to the factors used in the fMRI analysis.
Examples of questions that loaded on to the Emotion/External Eating Factor: ' If food tastes good to you, do you eat more than usual?' 'Do you have the desire to eat when you are irritated?'
Examples of questions that loaded on to the Self Restraint Factor: 'If you have put on weight, do you eat less than you usually do?'. 'Do you take into account your weight with what you eat?
Examples of Questions that loaded on to the Self Efficacy Factor involve asking if the participant is confident in following their dietary plan in a variety of situations such as when upset, alone, hungry etc.

	Emotion/External regulation	Self restraint	Self efficacy	Communalities
DEBQ-C- emotional	**.784**	.270	-.081	.694
DEBQ-C- restrained	-.108	**.658**	.358	.572
DEBQ-C- external	**.641**	.170	-.289	.523
Motivation Healthy Diet- Identified	-.457	**.679**	-.337	.784
Motivation Healthy Diet-extrinsic	.463	**.575**	.343	.663
Motivation Healthy Diet- amotived	**.742**	.186	.182	.619
Motivation Healthy Diet- intrinsic	-.507	**.658**	-.273	.765
Appraisal healthy eating	-.489	.020	**.612**	.614

Table E: One-tailed partial correlation coefficients between regression factor scores and adolescents report of their eating behaviour, and intake (controlling for age and parental education), split by group. N sizes for each analysis vary due to pairwise deletion of missing data to maximise power.

	Adolescent Regression Factor 1: Emotional/External regulation			Adolescent Regression Factor 2: Self Restraint			Adolescent Regression Factor 3: Self Efficacy			Total calories at lunch			Total calories at snack		
	CTL	OB	T2 DM	CTL	OB	T2 DM	CTL	OB	T2 DM	CTL	OB	T2 DM	CTL	OB	T2DM
Parent Regression Factor 1 Teaching and Modelling	.28	-.01	-.60 p=.019	.15	.48 p=.02	.44	-.20	.45 p=.027	.13	.48	-.37	-.46	.22	.24	-.006*
Parent Regression Factor 2 Emotive/External feeding	.33	.15	.64 p=.013	-.03	-.19	-.19	.47 p=.025	.03	.36	.18	-.19	.10	.13	.08	-.40
Parent Regression Factor 3 Restrictive Feeding	.41 p=.045	.08	.39	.30	.11	-.27	-.52 p=.013	.26	.26	.33	-.09	.38	-.38	.05	.47
Adolescent Regression Factor 1: Emotional/External regulation	-	-	-	-	-	-	-	-	-	-.06	-.12	.21	-.12	.20	.04
Adolescent Regression Factor 2: Self Restraint	-	-	-	-	-	-	-	-	-	.14	-.01	-.02	.04	-.04	-.01
Adolescent Regression Factor 3: Self Efficacy	-	-	-	-	-	-	-	-	-	.05	-.05	.15	.31	.02	-.003

CTL = healthy weight controls OB= obese T2DM= Type 2 DM. ** p<0.01

Table F: Areas of the brain where there is higher activation for food images compared to visually matched non-food images.

Contrast	Voxels	Z MAX	Max MNI co-ordinates X	Y	Z	Region
Healthy weight controls	45066	5.96	-42	-8	6	Left Insula
			40	6	-10	Left Insula
			64	-12	12	Central Opercular Cortex
			-42	-2	-2	Left Insula
			58	-14	28	Right PostCentral Gyrus
			-50	-56	46	Left Angular Gyrus
Obese	1976	4.05	-8	-68	36	Left Precuneus
			-14	-62	28	Left Precuneus
			-8	-36	8	Left Thalamus
			6	-32	6	Right Thalamus
			-12	-58	38	Left Precuneus
			12	-66	44	Right Precuneus
	1080	4.17	-46	-52	42	Left Angular Gyrus
			-44	-54	36	Left Angular Gyrus
			-54	-48	50	Left Supramaginal Gyrus
			-48	-46	34	Left Supramaginal Gyrus
			-60	-46	30	Left Supramaginal Gyrus
			-34	-60	34	Left Superior Lateral Occipital Cortex
	910	4.04	18	62	-10	Right Frontal Pole
			16	60	-14	Right Frontal Pole
			18	44	28	Right Superior Frontal Gyrus
			22	40	28	Superior Frontal Gyrus
			26	56	-16	Right Frontal Pole
			38	48	-6	Right Frontal Pole
Type 2 DM	5661	5.15	-4	-68	38	Left Precuneus
			-6	-68	34	Left Precuneus
			-4	-22	32	Left Posterior Cingulate
			-4	-18	24	Corpus Callosum
			4	-80	36	Right Cuneus
			6	-72	40	Right Precuneus
	5581	4.97	-4	46	0	Left Paracingulate Gyrus
			2	42	14	Right anterior Cingulate
			-4	38	8	Left Cingulate
			-4	52	8	Left Paracingulate Gyrus
			14	58	20	Right Frontal Pole
			14	62	-10	Right Frontal Pole
	4164	4.99	-56	-56	28	Left Angular Gyrus
			-52	-62	34	Left Superior Lateral Occipital Cortex
			-52	-50	28	Left Supramaginal Gyrus

			-46	-54	38	Left Angular Gyrus
			-34	-44	30	Left Supramaginal Gyrus
			-48	-48	34	Left Supramaginal Gyrus
	4132	4.83	64	-52	26	Right Angular Gyrus
			48	-44	30	Right Angular Gyrus
			48	-44	38	Right Angular Gyrus
			50	-44	42	Right Supramarginal Gyrus
			62	-28	-12	Right Medial Temporal Gyrus
			68	-24	-20	Right Medial Temporal Gyrus
	2485	4.32	30	20	-24	Right Orbito Frontal Cortex
			60	12	12	Right Inferior Frontal Cortex, pars opercularis
			40	8	-12	Right Insula
			36	12	-20	Right Insula
			56	10	6	Right Inferior Frontal Cortex, pars opercularis
			38	4	-6	Right Insula
	2418	4.62	-48	26	32	Left Medial Frontal Gyrus
			-40	50	0	Left Frontal Pole
			-46	16	40	Left Frontal Pole
			-26	12	28	Left Medial Frontal Gyrus
			-40	14	36	Left Medial Frontal Gyrus
			-28	10	38	Left Medial Frontal Gyrus

Table G: Areas of the brain where activation for food images (compared to non-food images) is correlated with BMI.

Contrast	Voxels	Z MAX	Max MNI co-ordinates			Region
			X	Y	Z	
Type 2 DM & Obese						
	2015	5.1	-38	-6	2	Left Insula
		4.85	-40	-54	42	Left Angular Gyrus
		4.69	-10	8	64	Left Superior Frontal Gyrus
		4.68	-24	58	-6	Frontal Pole
		4.6	-32	66	-2	Frontal Pole
		4.56	42	48	-6	Frontal Pole
	8012	4.26	60	6	8	Right Inferior Frontal Gyrus
		4.15	2	-36	20	Right Posterior Cingulate
		4.05	-14	-58	28	Left Precuneus
		4.01	-8	-68	36	Left Precuneus
		3.94	-4	-26	28	Left Cingulate
		3.92	58	18	6	Right Inferior Frontal Gyrus
	1922	4.18	54	-34	-2	Right Medial Temporal Gyrus
		4.15	60	-32	-6	
		3.96	56	-34	-8	
		3.96	52	-40	-4	
		3.94	54	-24	-10	
		3.66	62	-46	20	

Healthy weight controls & Obese						
	4141	3.98	-16	40	26	Left Medial Frontal Gyrus
		3.83	-12	42	42	Left Superior Frontal Gyrus
		3.8	-36	40	22	Left Medial Frontal Gyrus
		3.71	-10	6	64	Left Superior Frontal Gyrus
		3.59	2	2	66	R Superior Frontal Gyrus
		3.59	4	0	62	
	3204	3.91	-48	-48	36	Left Supramarginal Gyrus
		3.89	-50	-44	42	Left Supramarginal Gyrus, inferior Parietal
		3.88	-48	-42	38	Left Supramarginal Gyrus, Superior Parietal
		3.83	-44	-52	44	Left Supramarginal Gyrus, inferior Parietal
		3.55	-50	-14	14	Parietal Operculum
		3.48	-64	-14	24	Left Post Central Gyrus
	979	3.7	48	4	-2	Right Operculum/insula
		3.49	58	6	12	Right Inferior Frontal Gyrus
		3.44	60	6	8	Right Prefrontal Gyrus
		2.85	54	16	4	Right Inf Frontal Gyrus
		2.82	64	-10	20	
		2.81	64	22	14	
	943	3.68	0	-44	6	Corpus Callosum
		3.49	2	-40	26	Right Cingulate
		3.24	12	-48	22	Right Posterior Cingulate/precuneus
		3.18	-10	-34	22	Corpus Callosum
		3.04	6	-36	20	Corpus Callosum
		3.01	0	-30	28	Posterior Cingulate

Table H: Areas of the brain where activation for food images is higher for higher fat foods, compared to visually matched non-food images.

Contrast	Voxels	Z MAX	Max MNI co-ordinates			Region
			X	Y	Z	
Type 2 DM - Obese						
	1328	3.78	10	-74	30	Visual cortex
			6	-68	20	Right Superior Calacarine Sulcus
			-8	-70	18	Left Superior Calcarine Sulcus
			8	-78	34	Left Visual Cortex
			-10	-84	12	Left Calcarine Sulcus
			-14	-80	12	Left Calcarine Sulcus
	778	3.74	66	-24	-20	Right Medial Temporal Gyrus
			64	-36	4	Right Superior Temporal Gyrus
			60	-16	-20	Right Medial Temporal Gyrus
			64	-54	24	Right Angular Gyrus
			46	-72	42	Right Inferior Parietal
			64	-52	14	Right Angular Gyrus

Healthy weight controls -Obese						
	1845	4.71	-38	14	-2	Left Insula
			-44	-6	6	Left Operculum
			-58	32	-4	Left Orbito-Frontal Cortex
			-60	-22	12	Left Operculum
			-44	-2	-2	Left Insula
			-58	18	-4	Left Inferior Frontal Cortex
	1539		40	6	-10	Right Insula
			62	-24	36	Right Supramarginal Gyrus
			40	10	-8	Right insula
			62	-12	12	Right Operculum
			44	16	-16	Right insula
			70	-48	2	Right Medio Temporal Gyrus
